# Preoperative factors predicting the severity of BMD loss around the implant after Total hip Arthroplasty

**DOI:** 10.1186/s12891-021-04161-4

**Published:** 2021-03-19

**Authors:** Akira Morita, Naomi Kobayashi, Hyonmin Choe, Taro Tezuka, Shota Higashihira, Yutaka Inaba

**Affiliations:** 1 Department of Orthopaedic Surgery, Yokohama City University, 3-9 Fukuura, Kanazawa-ku, Yokohama 236-0004 Japan; 2 Department of Orthopaedic Surgery, Yokohama City University Medical Center, 4-57 Urafune-cho, Minami-ku, Yokohama 232-0024 Japan

**Keywords:** Total hip arthroplasty, Bone mineral density, Canal flare index, Zweymüller

## Abstract

**Background:**

Stress shielding after total hip arthroplasty (THA) leads to loss of bone mineral density (BMD) around the femoral implants, particularly in the proximal area. Loss of BMD around the implant is likely to occur within 1 year after THA; however, its severity depends on patient characteristics. This study evaluated preoperative factors correlated with the severity of zone 7 BMD loss after THA.

**Methods:**

This retrospective cohort study included 48 patients who underwent primary THA from October 2011 to December 2015. All patients underwent implantation of a Zweymüller-type femoral component without any postoperative osteoporosis medications. The objective variable was a change in zone 7 BMD after 1 year. Factors evaluated included age, body mass index, Japanese Orthopaedic Association score, Harris Hip Score, Canal Flare Index (CFI), and lumbar BMD on the frontal and lateral sides. Univariate and multivariate regression analyses identified factors correlated with loss of zone 7 BMD.

**Results:**

Univariate regression analysis identified CFI (*P* = 0.003) and preoperative lumbar BMD on the anterior-posterior (*P* = 0.003) and lateral (*P* < 0.001) sides as being correlated with loss of zone 7 BMD. Multivariate regression analysis identified CFI (*P* = 0.014) and lumbar BMD on the lateral side (*P* < 0.001) as being correlated independently with loss of zone 7 BMD.

**Conclusion:**

Lower preoperative lumbar BMD on the lateral side and lower CFI were correlated with zone 7 BMD loss after THA. Patients with these characteristics should be monitored carefully for severe BMD loss after THA.

## Introduction

Total hip arthroplasty (THA) is an established surgical method for patients with hip joint diseases such as osteoarthritis and osteonecrosis of the femoral head; THA results in stable long-term clinical outcomes, including pain relief and improvements in performance of activities of daily living (ADL) [1, 2]. However, THA can lead to a reduction in bone mineral density (BMD) around the implant, particularly in proximal parts such as Gruen’s zones 1 and 7 [3–5]. Although loss of BMD around the implant does not worsen clinical outcomes directly, it is associated with increased risk of periprosthetic fracture and late loosening [6, 7]. Thus, maintaining BMD around the implant provides stable long-term clinical outcomes.

The main cause of BMD loss around the implant is stress shielding, which is caused by changes in mechanical stress properties after implantation [4]. Several studies have assessed the ability of several drugs, including bisphosphonate and teriparatide, to prevent loss of BMD around implants following THA [8–12]. Because loss of BMD may also be associated with preoperative patient-specific factors, these findings suggest that drug interventions should be initiated immediately after THA, particularly in selected patients at higher risk of BMD loss. Therefore, this study sought to identify preoperative factors that predict the severity of BMD loss around implants after THA.

## Patients and methods

**Fig. 1:**
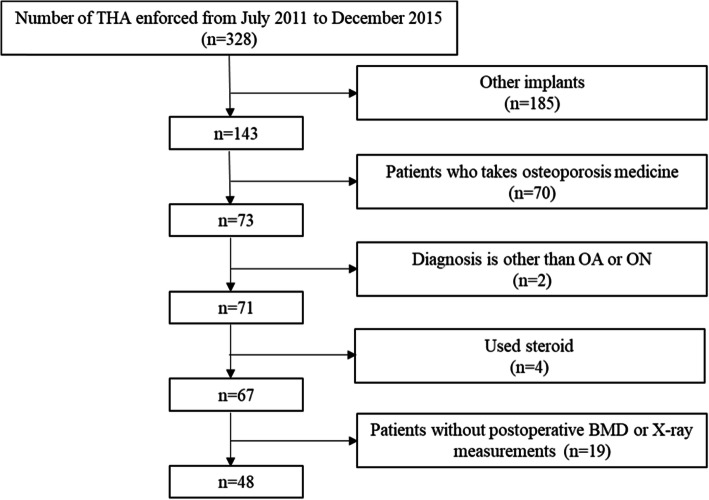
Flow chart showing the patient selection process

This retrospective cohort study evaluated patients diagnosed with osteoarthritis of the hip or osteonecrosis of the femoral head who underwent primary THA at Yokohama City University hospital from October 2011 to December 2015 (Fig. 1). Patients were excluded if they received non-target implants, were treated with drugs for osteoporosis or corticosteroids, or were diagnosed with a condition other than osteoarthritis of the hip or osteonecrosis of the femoral head. Patients were also excluded if not all data, including BMD and X-rays, were available 1 year after surgery. Preoperative patient activity levels were evaluated by measuring the activity score, the Japanese Orthopaedic Association (JOA) hip score, and the Harris Hip Score (HHS).

**Fig. 2:**
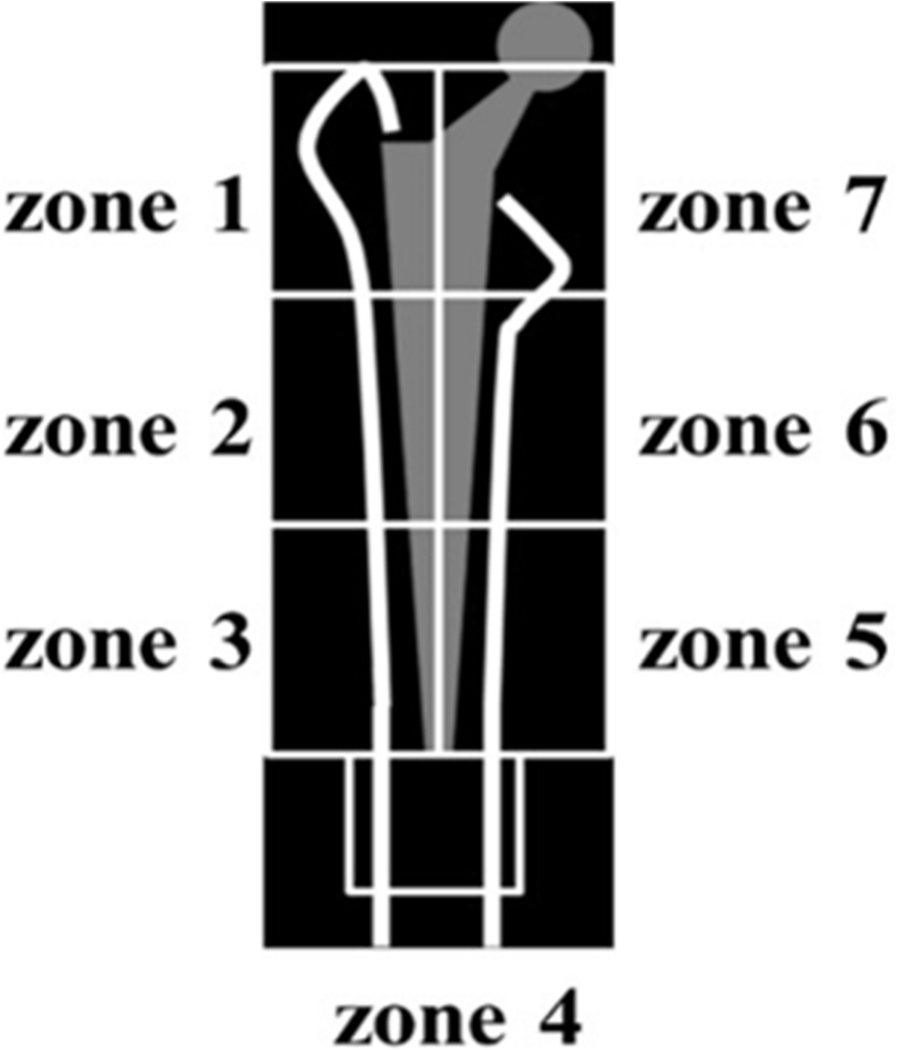
Gruen’s zone classification

All patients were operated on using a direct lateral approach and all received the same cementless femoral component of a Zweymüller-type stem (SL-PLUS MIA, Smith and Nephew, Inc. Memphis, TN), a cementless acetabular component (REFLECTION, Smith and Nephew, Inc.), and a cross-linked polyethylene liner (XLPE liner, Smith and Nephew, Inc.). All patients began using a wheelchair on the first postoperative day, all started gait exercises with full weight bearing as soon as possible thereafter. Dual-energy X-ray absorptiometry (DEXA) (QDR 2000, Hologic, Waltham, MA) was used to measure baseline periprosthetic BMD 1 week after THA, followed by subsequent measurements at 1-year intervals. Regions of interest (ROIs) were centered on the periprosthetic zones described by Gruen (Fig. 2). At 1 week pre-surgery, DEXA was used to measure the BMD of the lumbar (L) vertebrae (L2 to L4) in the anterior-posterior (AP) and lateral directions.

**Fig. 3:**
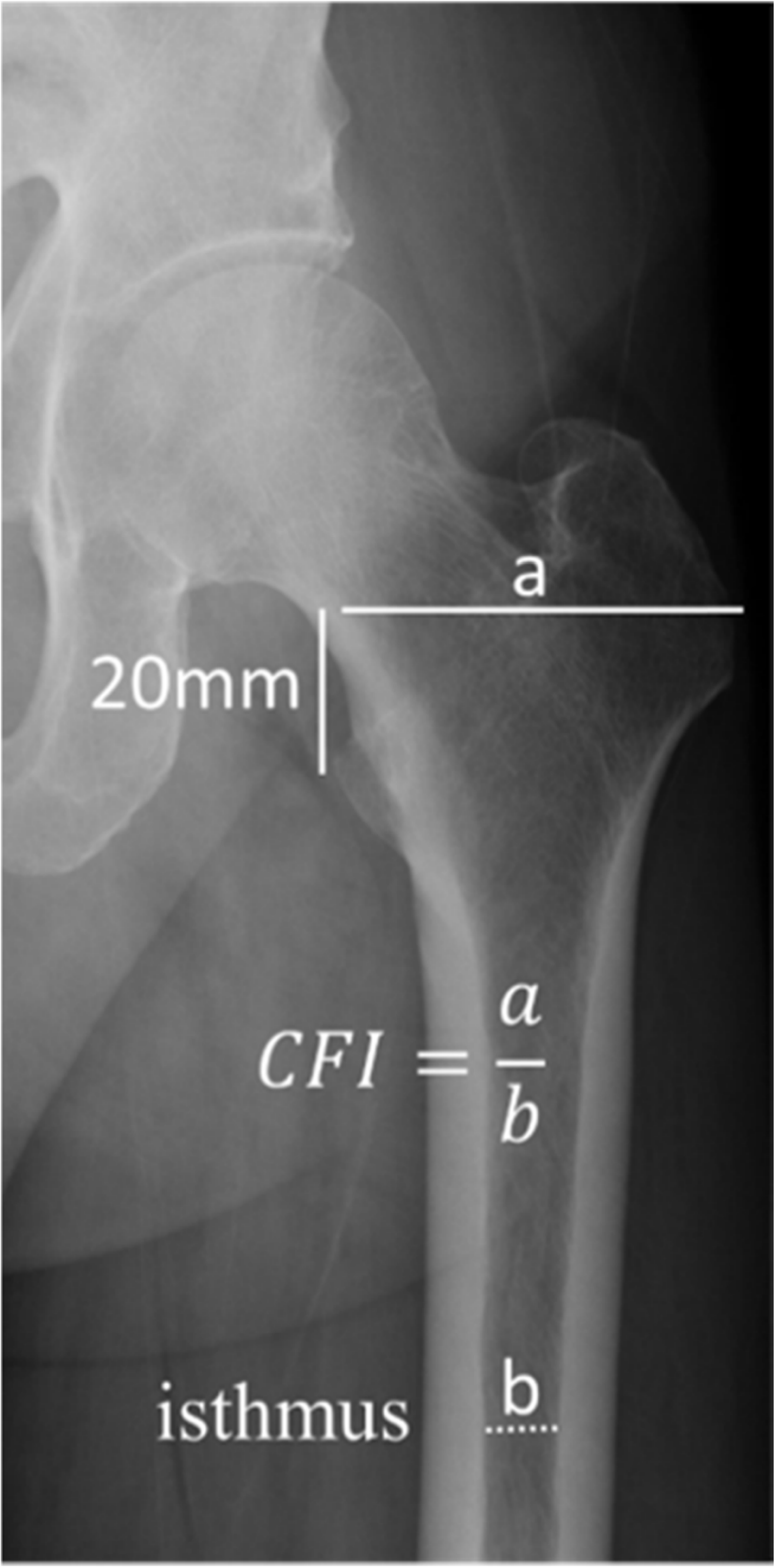
Canal flare index

The primary outcome variable was a change in zone 7 BMD 1 year after THA; the secondary outcome variable was a change in BMD in zones 1–6. Explanatory variables included age, body mass index (BMI), the preoperative Japanese Orthopaedic Association (JOA) score, the Harris Hip Score (HHS), the Canal Flare Index (CFI) (Fig. 3), and lumbar BMD on the AP and lateral sides.

### Statistical analysis

To analyze the factors that periprosthetic loss of BMD after THA, a regression analysis was performed. At first, a univariate regression analysis was performed with the following factors as explanatory variables: age, BMI, JOA score, HHS, CFI, lumbar BMD on the AP side and lateral side. Furthermore, variables with *p*-values < 0.05 were populated for regression analysis as a multivariate assessment and entered into the final equation. Standardized regression coefficients (β) and associated p-values were determined to assess statistical significance (*p* < 0.05). Univariate regression analysis and multivariate regression analysis analyses were performed using SPSS II software (SPSS Japan, Inc., Tokyo, Japan).

## Results

Of the 328 patients who underwent primary THA from October 2011 to December 2015, 280 were excluded (185 who received non-target implants, 70 who were taking medications for osteoporosis, two who were diagnosed with a condition other than osteoarthritis of the hip or osteonecrosis of the femoral head, four who were taking steroids, and 19 for whom data were missing (e.g., results of BMD measurements and X-rays)).

**Fig. 4:**
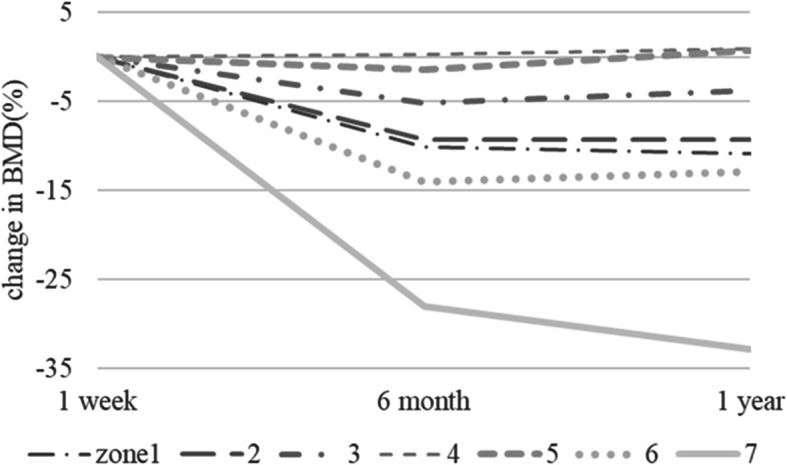
Time course of percent change in BMD from baseline in each Gruen zone over 1 year

**Fig. 5:**
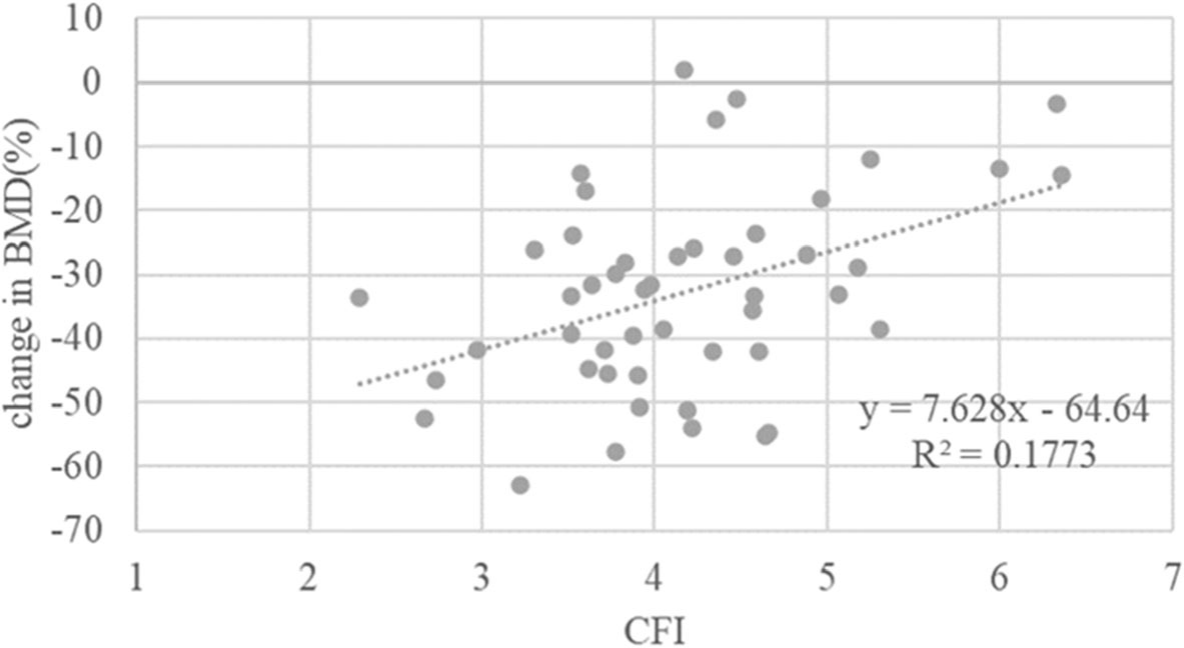
Scatter plot showing the relationship between the percentage change in zone 7 BMD and CFI from baseline to 1 year post-surgery (R^2^ = 0.177, r = 0.421, *P* = 0.003)

**Fig. 6:**
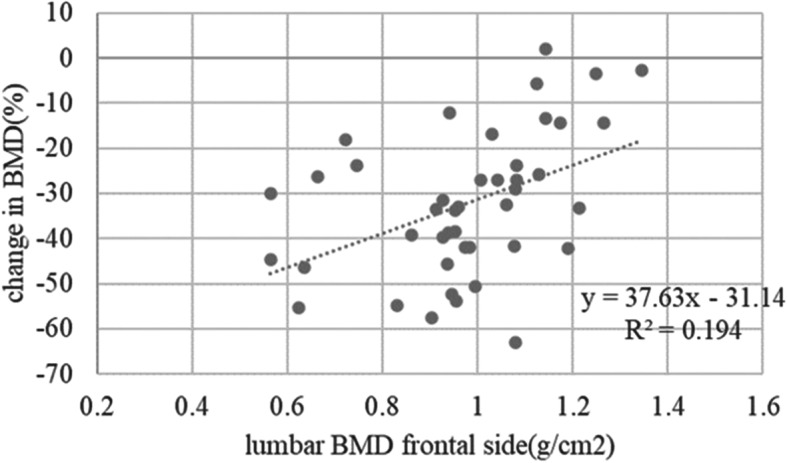
Scatter plot showing the relationship between the percentage change in zone 7 BMD and preoperative lumbar BMD on the frontal side from baseline to 1 year post-surgery (R^2^ = 0.194, r = 0.440, *P* = 0.003)

**Fig. 7:**
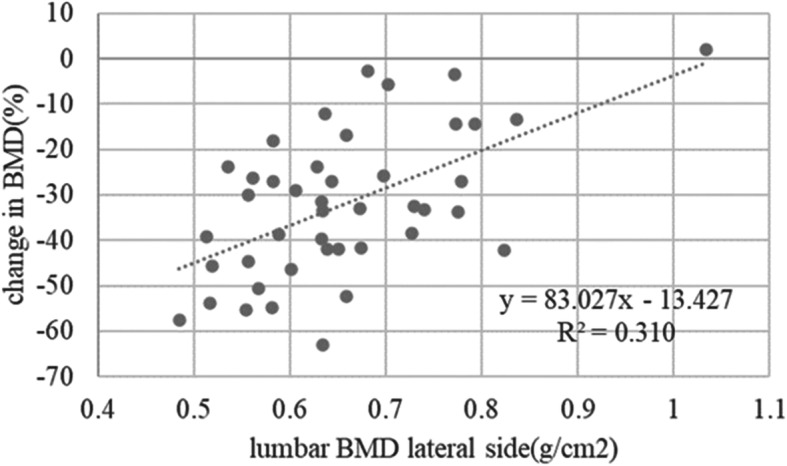
Scatter plot showing the relationship between the percentage change in zone 7 BMD and preoperative BMD on the lateral side from baseline to 1 year post-surgery (R^2^ = 0.310, r = 0.557, P < 0.001)

The mean (± standard deviation) age of the 48 enrolled patients was 64.0 ± 12.0 years and the mean BMI at the time of surgery was 24.0 ± 4.2 kg/m^2^. Table 1 shows the demographic and clinical characteristics of the cohort, and Fig. 4 shows the periprosthetic changes in BMD for each zone at 1 year post-surgery. Relative to baseline, the mean percentage changes in BMD for zones 1–7 at 1 year were − 10.9 ± 9.7, − 9.3% ± 11.5, − 3.7% ± 8.6%, + 0.9% ± 5.5%, + 0.8% ± 6.1%, − 12.9% ± 12.8%, and − 32.8% ± 15.3%, respectively. Table 2 shows the results of univariate regression analysis of the association between changes in BMD at zones 1–7 and exploratory variables. Scatter plots showed that the percentage change in zone 7 BMD from baseline to 1 year post-surgery correlated with preoperative CFI (R^2^ = 0.177, *P* = 0.003) (Fig. 5), preoperative lumbar BMD on the AP side (R^2^ = 0.194, *P* = 0.003) (Fig. 6), and preoperative lumbar BMD on the lateral side (R^2^ = 0.310, *P* < 0.001) (Fig. 7). Multivariate regression analysis showed that the percentage change from baseline in zone 2 correlated with lumbar BMD on the AP side (β = 0.344, *P* = 0.024) (adjusted R^2^ = 0.119). In zone 5, it correlated with age (β = 0.344, *P* = 0.012) and BMI (β = − 0.293, *P* = 0.032) (adjusted R^2^ = 0.196); in zone 6 it correlated with lumbar BMD on the lateral side (β = 0.357, *P* = 0.019) (adjusted R^2^ = 0.106); and in zone 7 it correlated with CFI (β = 0.322, *P* = 0.014) and lumbar BMD on the lateral side (β = 0.48, *P*<0.001) (adjusted R^2^ = 0.408) (Table 3).

**Table 1:** Demographic and clinical characteristics of the 48 patients included in the study

Variable	Mean ± SD
Age (yr)	64.0 ± 12.0
Male/Female	9/39
Body Mass Index (kg/m^2^)	24.0 ± 4.2
Japanese Orthopaedic Association score	52.0 ± 14.0
Harris Hip Score	51.8 ± 15.4
Canal Flare Index	4.2 ± 0.8
Lumbar BMD on the AP side (g/cm^2^)	1.0 ± 0.2
Lumbar BMD on the lateral side (g/cm^2^)	0.7 ± 0.1

**Table 2:** Univariate regression of associations between changes in BMD in each zone and exploratory variables*

Variables	Zone 1	Zone 2	Zone 3	Zone 4	Zone 5	Zone 6	Zone 7
**Age (yr)**	r = 0.174	r = 0.270	r = 0.143	r = 0.073	**r = 0.382**	r = 0.128	r = 0.172
	*P* = 0.238	*P* = 0.063	*P* = 0.333	*P* = 0.622	***P*** **= 0.007**	*P* = 0.387	*P* = 0.242
**Body Mass Index (kg/m**^**2**^**)**	r = −0.102	r = 0.021	r = 0.045	r = 0.043	**r = − 0.337**	r = 0.034	r = − 0.063
	*P* = 0.491	*P* = 0.886	*P* = 0.760	*P* = 0.770	***P*** **= 0.019**	*P* = 0.818	*P* = 0.672
**Japanese Orthopaedic Association score**	r = 0.153	r = 0.240	r = 0.090	r = −0.064	r = 0.104	r = −0.050	r = 0.223
	*P* = 0.306	*P* = 0.104	*P* = 0.548	*P* = 0.667	*P* = 0.487	*P* = 0.736	*P* = 0.132
**Harris Hip Score**	r = 0.041	r = 0.108	r = −0.019	r = 0.014	r = 0.051	r = −0.139	r = 0.134
	*P* = 0.782	*P* = 0.471	*P* = 0.901	*P* = 0.924	*P* = 0.736	*P* = 0.351	*P* = 0.369
**Canal Flare Index**	r = 0.108	**r = 0.294**	r = 0.243	r = 0.026	r = 0.045	r = 0.189	**r = 0.421**
	*P* = 0.467	***P*** **= 0.043**	*P* = 0.096	*P* = 0.859	*P* = 0.761	*P* = 0.198	***P*** **= 0.003**
**Lumbar BMD on the AP side (g/cm**^**2**^**)**	r = 0.006	**r = 0.344**	r = 0.157	r = −0.135	r = −0.190	r = 0.178	**r = 0.440**
	*P* = 0.968	***P*** **= 0.024**	*P* = 0.314	*P* = 0.389	*P* = 0.223	*P* = 0.252	***P*** **= 0.003**
**Lumbar BMD on the lateral side (g/cm**^**2**^	r = 0.066	**r = 0.335**	r = 0.165	r = 0.109	r = 0.080	**r = 0.357**	**r = 0.557**
	*P* = 0.672	***P*** **= 0.028**	*P* = 0.291	***P*** = 0.487	*P* = 0.609	***P*** **= 0.019**	***P*<0.001**

*Significant values are shown in bold

**Table 3:** Multivariate regression of associations between changes in BMD in each zone and exploratory variables

	Variables	Regression coefficient	β	P-value	Adjusted R^**2**^
**Zone 2**					0.119
	Lumbar BMD on the AP side (g/cm^2^)	21.958	0.344	0.024	
**Zone 5**					0.196
	Age (yr)	0.180	0.344	0.012	
	Body Mass Index (kg/m^2^)	−0.428	−0.293	0.032	
**Zone 6**					0.106
	Lumbar BMD on the lateral side (g/cm^2^)	44.011	0.357	0.019	
**Zone 7**					0.408
	CFI	5.741	0.322	0.014	
	Lumbar BMD on the lateral side (g/cm^2^)	71.653	0.480	<0.001	

## Discussion

This retrospective cohort study investigated preoperative factors correlated with periprosthetic BMD loss after THA. Multivariate regression analysis showed that preoperative CFI and lumbar BMD on the lateral side correlated with BMD loss in zone 7. These findings emphasize the importance of monitoring patients for severe BMD loss after THA, particularly patients with lower BMD in the lumbar region and a stovepipe-shaped proximal femur.

BMD loss around the implant is common after THA [13, 14]. The most important cause is stress shielding, which is influenced mainly by stem design. A comparison of patients undergoing THA with the Zweymüller stem or fit-and-fill stem implants found that mechanical stress and zone 7 BMD loss around the implant were lower in those receiving the Zweymüller stem [4].

Loss of BMD may also be associated with patient-specific or operative factors. For example, excessive stem anteversion mismatched with anatomical canal anteversion results in contact between the stem point and the cortical bone in the distal portion, thereby affecting proximal periprosthetic zone 7 BMD loss after THA [15, 16]. An investigation of postoperative zone 7 BMD in groups of patients with normal preoperative lumbar BMD and patients with osteopenia and osteoporosis found that BMD loss was significantly higher in the osteopenia and osteoporosis groups than in the normal group [17], indicating that periprosthetic BMD loss is associated with bone quality as well as stress shielding [18]. In this study, we confirmed that CFI correlates with zone 7 BMD loss. Although it was less relevant in univariate regression analysis, the adjusted R^2^ obtained from multivariate regression analysis was 0.408 when including both CFI and lumbar BMD on the lateral side. Thus, the equation might be helpful for predicting loss of BMD in zone 7.

Because loss of BMD in the proximal femur, particularly zone 7, is likely to be lower than that in other zones and occurs within 1 year postoperatively [17], it is important to take steps to prevent it. Many drugs are used to prevent loss of BMD around the implant after THA; one example is bisphosphonate [10, 11, 19–24]. Moreover, bisphosphonate are associated with a lower risk of aseptic revision in patients undergoing primary THA for osteoarthritis [21]. However, long-term continuous treatment with bisphosphonate is associated with atypical periprosthetic fractures [23, 24]. A study of a large US cohort reported that the periprosthetic fracture rate following primary THA was 1.1% [23]. Data from the Swedish hip registry suggest a rate of 0.64% over 10 years [24]; the latter also showed that bisphosphonate are associated with a higher risk of periprosthetic fracture in younger patients with normal bone quantity [24]. Another effective agent that can prevent loss of BMD around the implant is teriparatide. A randomized controlled trial found that teriparatide and alendronate were equally effective for preventing zone 7 BMD loss [11]. Moreover, switching from teriparatide to alendronate is effective [12]. Although several drugs prevent loss of BMD around the implant after THA, care is necessary regarding the side effects and economic burden of these agents.

The shape of the femoral medullary cavity is related to loss of BMD around the implant. A previous study shows that postoperative zone 7 BMD is significantly lower in stovepipe-shaped than in champagne-flute-shaped cavities when using taper-wedge-type stems [25]. The stovepipe-shaped type of medullary cavity has a small CFI, making these results similar to those of the present study. By contrast, a comparison of postoperative changes in BMD in any zone with respect to three types of medullary cavity (stovepipe, normal, and champagne-flute shaped) after implantation of a Zweymüller-type stem found no statistically significant difference in relative changes between the groups [26]. Here, we found that patients with low CFI, such as those with a stovepipe-shaped medullary cavity, showed a greater reduction in postoperative zone 7 BMD than patients with high CFI, such as those with a champagne-flute-shaped cavity.

Regarding other zones (1–6), univariate regression analysis showed that CFI and lumbar BMD correlated with BMD changes in zone 2. This result is similar to that observed for zone 7; thus, BMD loss in the proximal area may be correlated with CFI and lumbar BMD. By contrast, we found a correlation between age and changes in BMD in zone 5. When using the Zweymüller stem, a wide femoral canal and thin cortical bone correlate positively with increased bone density below the stem, resulting in hypertrophy of the cortical bone in the distal femur [27]. The canal flare index and cortical index for both sexes decreases with age [28], which may explain the correlation between age and changes in BMD in zone 5. In addition, we also found a weak correlation between BMD changes in zone 5 and BMI. Hayashi et al. [29] found no correlation between BMI and periprosthetic BMD when using a cementless triple tapered stem. This difference may be due to the implants used. Nevertheless, these zonal losses of BMD (except those in zone 7) are mild and may not be clinically important.

Finally, there is no clear evidence that BMD loss in zone 7 is directly related to lower survival rates of implants used for THA. However, in terms of preventing periprosthetic fractures, maintaining BMD around the implant is desirable. Therefore, in addition to implant design, it may be desirable to administer osteoporosis-preventing drugs after surgery, particularly in cases with lower lumbar BMD and lower CFI.

### Study limitations

This study has several limitations. First, we investigated only patients with Zweymüller-type stems. Studies of other stem types, such as taper-wedge stems, may yield different results. Second, the total number of evaluated subjects was small, as many subjects had to be excluded.

## Conclusion

Lower preoperative lumbar BMD on the lateral side and lower CFI correlated with zone 7 BMD loss at 1 year post-THA. Patients at risk of BMD loss may benefit from pre- or postoperative drug treatment to prevent this.

## Data Availability

The datasets used and/or analyzed during the current study are available from the corresponding author upon reasonable request.

## Abbreviations

THA: Total hip arthroplasty
BMD: Bone mineral density
BMI: Body mass index
JOA: Japanese Orthopaedic Association
HHS: Harris Hip Score
CFI: Canal Flare Index
ADL: Activities of daily living
DEXA: Dual-energy X-ray absorptiometry
ROIs: Regions of interest
AP: Anterior-posterior

## Declarations

All clinical studies were conducted in accordance with ICMJE guidelines.
